# Skin healing and scale regeneration in fed and unfed sea bream, *Sparus auratus*

**DOI:** 10.1186/1471-2164-12-490

**Published:** 2011-10-07

**Authors:** Florbela A Vieira, Silvia F Gregório, Serena Ferraresso, Michael AS Thorne, Rita Costa, Massimo Milan, Luca Bargelloni, Melody S Clark, Adelino VM Canario, Deborah M Power

**Affiliations:** 1 Comparative and Molecular Endocrinology Group, Centre for Marine Sciences (CCMAR), Universidade do Algarve, Campus de Gambelas, 8005-139 Faro, Portugal; 2 Dipartimento di Sanità Pubblica, Patologia Comparata e Igiene Veterinaria, Università di Padova, 35020 Legnaro, Italy; 3 British Antarctic Survey, Natural Environment Research Council, High Cross, Madingley Road, Cambridge, CB3 0ET, UK

## Abstract

**Background:**

Fish scales are an important reservoir of calcium and phosphorus and together with the skin function as an integrated barrier against environmental changes and external aggressors. Histological studies have revealed that the skin and scales regenerate rapidly in fish when they are lost or damaged. In the present manuscript the histological and molecular changes underlying skin and scale regeneration in fed and fasted sea bream (*Sparus auratus*) were studied using a microarray 3 and 7 days after scale removal to provide a comprehensive molecular understanding of the early stages of these processes.

**Results:**

Histological analysis of skin/scales revealed 3 days after scale removal re-epithelisation and formation of the scale pocket had occurred and 53 and 109 genes showed significant up or down-regulation, respectively. Genes significantly up-regulated were involved in cell cycle regulation, cell proliferation and adhesion, immune response and antioxidant activities. 7 days after scale removal a thin regenerated scale was visible and only minor changes in gene expression occurred. In animals that were fasted to deplete mineral availability the expression profiles centred on maintaining energy homeostasis. The utilisation of fasting as a treatment emphasised the competing whole animal physiological requirements with regard to barrier repair, infection control and energy homeostasis.

**Conclusions:**

The identification of numerous genes involved in the mitotic checkpoint and cell proliferation indicate that the experimental procedure may be useful for understanding cell proliferation and control in vertebrates within the context of the whole animal physiology. In response to skin damage genes of immune surveillance were up-regulated along with others involved in tissue regeneration required to rapidly re-establish barrier function. Additionally, candidate fish genes were identified that may be involved in cytoskeletal re-modelling, mineralization and stem cells, which are of potential use in aquaculture and fish husbandry, as they may impact on the ability of the fish to produce structural proteins, such as muscle, efficiently.

## Background

In vertebrates the skin performs many functions, not least of which is protection from the external environment. It has a relatively well conserved organisation, composed of the epidermis, dermis, and hypodermis, but is obviously adapted to the habitat and environmental challenges that a particular species faces. In aquatic organisms, such as fish, the skin is also an important osmoregulatory organ and the scales act as a reservoir of minerals [1-5]. The living non keratinized epidermis and scales are a functional specialisation of teleost skin and the latter structures are dermal skeletal elements which form after metamorphosis in juvenile fish [6]. The scales in most teleosts are classified as elasmoid and consist of an external calcified layer and a thicker, partially calcified basal plate composed of closely packed type I collagen fibrils [7,8]. The basal plate overlays elasmoblasts (scale forming cells) and resorption involves the action of osteoclasts (scale resorbing cells) [9-13].

Scale removal in fish involves the loss of epidermal cells, scales and the superficial dermis. Such skin wounds heal rapidly in fish and the skin surface is quickly covered by mucus and re-epithelization from the wound margin occurs within a few hours [1,14]. Moreover, within a few weeks a new scale with the size and characteristics of a mature scale is completely re-grown [8,15]. This process of regeneration has been divided into four stages; starting with re-epithelization and the differentiation of scale-forming cells (day 1-2), followed by rapid production of external layer matrix (days 3-5), the production of basal-plate matrix (days 6-14) and finally partial mineralization of the basal plate (very intense by days 14-28) [8]. To date most studies on scale formation and/or regeneration have focussed on morphology [7,16], with a limited understanding of the associated molecular basis, which is restricted to single gene studies. For example, co-expression of the estrogen receptor 2a (*esr2a*), apolipoprotein Eb (*apoeb*) and sonic hedgehog (*shh*) has been linked to cell proliferation, differentiation and metabolic activity of the zebrafish epidermis in fin buds and growing scales [17-19]. The ectodysplasin-A-receptor (EDAR) has been shown to be required for scale initiation and may also be involved in the cross-talk between the epidermal basal cells and the differentiating scale-forming cells in medaka (*Oryzias latipes*) [20]. Moreover, recently MMP-2 and MMP-9 were shown to have a role in scale regeneration in zebrafish [21]. Removal of scales damages a key barrier of the innate immune system and consequently provokes an inflammatory response and activation of the processes associated with healing and skin and scale re-growth [22,23].

Along with their protective role, scales also provide a readily mobilized reservoir of calcium in periods of high-calcium demand and contribute to whole organism calcium homeostasis [2,3,24,25]. Calcium in its soluble form is essential for cellular enzyme activities, nerve and muscle function and is a significant component of skeletal architecture including bone and scales. Its levels are tightly regulated [26-28]. Calcium in bones and scales is closely associated with phosphorus in the form of hydroxyapatite. Hence regeneration and repair of scales not only affects calcium levels, but also those of phosphorus, which like calcium, is essential for bone integrity and has numerous other essential cellular functions [29].

The aim of this study is to gain an understanding of the molecular basis of scale regeneration in a teleost fish, the gilthead sea bream (*Sparus auratus*, Linnaeus 1758). In particular, calcium and phosphorus are essential for the calcified matrix of forming scales and the effect on regeneration of manipulating minerals via food availability was assessed. Scale regeneration was monitored by analysing temporal changes in skin/scale morphology and modifications in the transcriptome determined using a sea bream specific oligo microarray.

## Results and Discussion

The experiments represented three treatments: animals with scales removed (WS), fasted animals (ST) and fasted animals with scales removed (STWS); and control animals (N). The sea bream scale regeneration process was evaluated at two time points, day 3 and day 7 after scale removal. Food deprivation was employed as a treatment to reduce the transcriptome associated with cellular/tissue metabolism and modify whole animal mineral homeostasis and in this way cause a relative amplification in the gene expression signals generated as a result of the cellular response to scale removal. There were no evident signs of stress, no mortality occurred during the experimental trial and no overt infections were evident. Sea bream from which food was withheld failed to increase in length and weight during the experiment compared to those that were fed irrespective of the presence or absence of scales. The good condition of the animals was substantiated by measurements of plasma components. Lactate and glucose were the plasma components measured to investigate the condition of animals.

### Morphology of sea bream skin/scales

**Figure 1:**
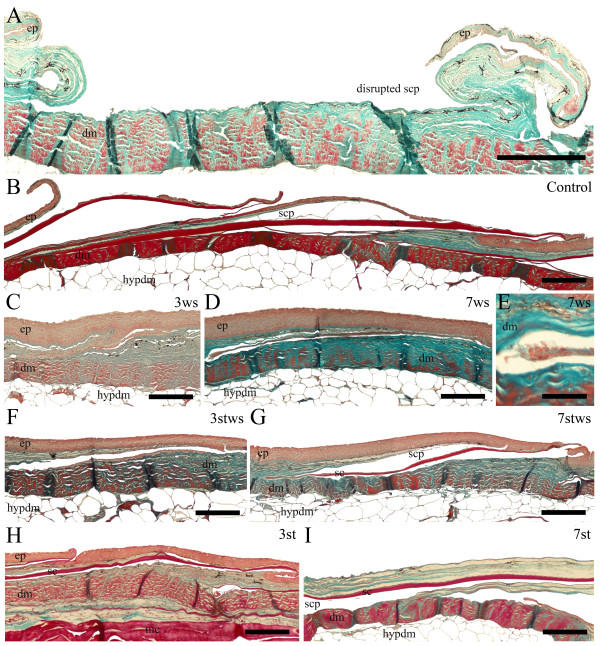
**Longitudinal transverse sections (5 μm) of sea bream skin from each of the experimental groups**. A) Removal of scale; B) N (control); C) 3WS (without scales day 3); D) 7WS (without scales day 7); E) amplification of 7WS (without scales day 7); F) 3STWS (fasted without scales day 3); G) 7STWS (fasted without scales day 7); H) 3ST (fasted day 3) and I) 7ST (fasted day 7) stained with Masson's Trichrome. The posterior region of the scale is orientated to the right. Connective tissue is stained green and mineralized and collagen-rich tissues are stained bright red. Sc - scale; scp - scale pocket; ep - epidermis; dm - dermis; hypdm - hypodermis and mc - muscle are indicated. Scale bars: A,B,C,D,F,G,H - 200 μm; E - 50 μm.

Transverse sections of skin from all the experimental groups at both time points (days 3 and 7) of the experiment were analysed (Figure 1). Sea bream skin had the typical organisation of teleost skin and was composed of three well defined layers: the epidermis, dermis and hypodermis which overlaid a fat layer that varied in thickness. The scales were each enclosed within a scale pocket and were composed of a mineralized external layer and a partially mineralized basal plate. The scale pocket was localized in the superficial dermis and projected into and was covered by a thin layer of epidermis (Figure 1B, H and 1I). Removal of the scales damaged the epidermis, dermis and scale pocket, the latter two tissues became exposed to the ambient water and the epidermis which remained attached to the dermis hung loose (Figure 1A). The ontogeny of the regenerative response was similar in all sea bream. Histology of the day 3 samples revealed a rapid repair process, with the epidermis already re-established and the enclosed scale pocket without a scale was visible in the dermis (Figure 1C and 1F). Hence within 3 days, the animals had re-established their external barrier and protection to the environment and 7 days after scale removal a thin regenerated scale was visible (Figure 1D, E and 1G). From a morphological perspective the regeneration process in sea bream was similar to that described in the cichlid *Hemichromis bimaculatus *[1,30] and also in zebrafish and goldfish [8,18].

### Plasma analyses

No significant difference in plasma glucose or plasma lactate (frequently used as indicators of physiological stress) was observed between any of the experimental groups analyzed on day 3 of the experiment (Table 1). Plasma glucose and lactate levels were both within the normal range for fish with normal metabolism and not suffering from stress [31-33]. No significant difference in total plasma calcium was found between any of the experimental groups and the control (Table 1), with concentrations in the normal range for intact animals of this species [34]. Plasma phosphorus levels also varied within normal levels (Table 1). However, a significant (p < 0.05) reduction in plasma phosphorus was measured in animals without scales which were fasted (2.685 mmol/L ± 0.225) in relation to the fed animals without scales (3.755 mmol/L ± 0.234). Nutrient depletion will amplify the effects of scale removal as both will cause increased mineral requirements by the fish in order to maintain whole body calcium and phosphorus homeostasis. Phosphorus is mainly obtained via the diet, whilst calcium can be obtained from both the diet and seawater [35]. Hence when fish are deprived of food the requirement for these minerals will be evidenced first via the phosphorus measurements (as observed here) that probably acts as an indicator of enhanced calcium mobilization from sea water by the fish.

**Table 1:** Sea bream plasma parameters concentrations measured for the different groups at day 3.

Group	**Calcium (mmol.L**^**-1**^**)**	**Phosphorus (mmol.L**^**-1**^**)**	**Glucose (mmol.L**^**-1**^**)**	**Lactate (mmol.L**^**-1**^**)**
3N	2.984 ± 0.158	3.256 ± 0.077	4.539 ± 0.370	2.562 ± 0.343
3WS	3.377 ± 0.116	3.755 ± 0.234	4.542 ± 0.573	2.821 ± 0.352
3ST	3.047 ± 0.113	3.349 ± 0.182	5.631 ± 0.628	3.154 ± 0.228
3STWS	2.986 ± 0.069	2.685 ± 0.225	5.131 ± 0.281	2.779 ± 0.379

Values correspond to mean values ± SEM from each group (individual biological replicates of n = 8).

### Molecular analyses

**Figure 2:**
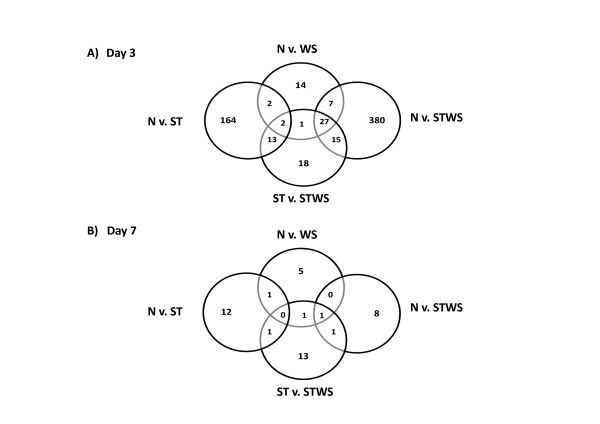
**Venn diagrams of up-regulated genes**. The number of up-regulated genes is represented for each of the comparisons made for both day 3 (A) and day 7 (B) of the experiment. Both genes with known and unknown function were included. N stands for control fish in normal conditions; WS stands for fish fed normal ration levels but with ~60-70% of scales removed; ST stands for fasted fish and STWS stands for fasted fish with ~60-70% of scales removed.

Although the sea bream oligo-array had been previously annotated [36], the sequences of the oligos used in the microarray were reanalysed in order to take advantage of the recent large increase in molecular data available for teleosts. Of the 39,379 oligo probes on the array, 16,025 (40.6%) showed significant match similarity to a known protein in uniprot database (1e^-10^) [37]. To facilitate the understanding of the underlying cellular processes of epidermis and scale regeneration, a number of comparisons were carried out at days 3 and 7 after scale removal. Control animals (N) were compared with fed animals without scales (WS), fasted animals (ST) and fasted animals without scales (STWS). To specifically dissect out the enhanced effects of scale removal under conditions of nutrient depletion, an additional comparison of fasted animals (ST) with fasted animals which had had scales removed (STWS) was made. Table 2 shows the numbers of differentially regulated genes under these comparisons, with the major effect shown for the fasted vs. fasted without scales analysis (additional file 1). It is clear that within the skin, the response to scale removal is rapid and of short duration (even under food deprivation). To obtain a clear overview of the transcripts with a conserved response between the different comparisons Venn diagrams were generated for the up-regulated genes at both day 3 and 7 (Figure 2). For example of the 53 up-regulated genes in fish without scales compared to the control, 27 were also significantly up-regulated in unfed fish without scales compared to control and fasted fish.

**Table 2:** Microarray results in terms of number of probes differentially expressed between the different experimental groups.

	N compared with			ST compared with STWS
**Day 3**	**WS**	**ST**	**STWS**	
Up-regulated	53	181	66	429
Down-regulated	109	71	127	340
**Total**	**162**	**252**	**193**	**769**
				
**Day 7**				
Up-regulated	8	14	17	10
Down-regulated	4	31	31	11
**Total**	**12**	**45**	**48**	**21**

By day 7 there were much reduced levels of differential expression between groups with only 49 up-regulated probes compared to 729 up-regulated probes over the four comparisons at day 3. The considerable difference in gene expression found between day 3 and 7 reflects the rapid repair response and temporally different processes that accompany skin repair. Since most of the expression changes took place within three days and resulted in the differential expression of a considerable number of probes, an initial overview of the major processes involved was conducted before a more detailed gene-by-gene analysis.

### Overview of expression profiles via GO enrichment and Ingenuity pathway analysis

Microarray probes were classified according to their Gene Ontology terms (GO ID) in order to determine whether particular biological processes were enriched in response to the different treatments. Overall, 25.3% of the probes were associated with at least a GO term. The most represented Biological Processes on the microarray were "cellular processes" (GO:0009987) (24%), "regulation of biological process" (GO:0050789) (16%), "response to stimulus" (GO:0050896) (13%) and "multicellular organismal development" (GO:0007275) (12%) (Additional file 2). Interestingly, when GO enrichment analysis was performed on differentially expressed genes from different comparisons, no particular Biological Process term was enriched amongst the up-regulated gene lists with the exception of "cellular processes" between the STWS and the ST groups. The down-regulated gene lists revealed a significant reduction in metabolic processes, indicating that the animals were repartitioning their translation machinery away from normal housekeeping functions towards repair and regeneration (data not shown). This conclusion is substantiated by the Ingenuity pathway analysis software (IPA) results which identified the main molecular and cellular biological functions that were significantly affected (Table 3) and also which physiological systems with regard to development and function were involved (Additional file 3). The IPA top networks for all the comparisons which included animals with scales removed produced matches to cancer, indicating that genes which have been implicated in non-controlled cell proliferation in human may be involved in normal cellular proliferation in fish skin. Not surprisingly the top networks also included those involved in the cell cycle, cellular growth and proliferation, and biological processes included tissue/organ development and morphology and haematopoiesis. Lipid metabolism was one of the most significant functions represented in IPA in the fasted fish indicating the effects of nutrient depletion on the general metabolism of the animals. This finding was substantiated in the STWS comparisons, which also included networks involved in vitamin and mineral metabolism (Table 3, Additional file 3). The Ingenuity results, whilst providing an overview of the main cellular processes affected in the experiments, provide more detail than the simplified GO enrichment analyses and link in far more directly to analysis of individual genes and their putative functional identification.

**Table 3:** Top three network functions obtained by IPA for the differentially expressed genes of each comparison 3 days after scale removal.

Group	Top Network Functions	Score	Focus Molecules
**Control v. Without scales (WS)**			

	Small Molecule Biochemistry, Genetic Disorder, Metabolic Disease	34	17
	Cell Morphology, Cancer, Cell Cycle	29	15
	Cell-To-Cell Signaling and Interaction, Cellular Movement, Tissue Development	26	13

**Control v. Starved (ST)**			

	Lipid Metabolism, Nucleic Acid Metabolism, Small Molecule Biochemistry	46	22
	Cellular Development, Cellular Growth and Proliferation, Connective Tissue Development and Function	31	16
	Cell-To-Cell Signaling and Interaction, Hematological System Development and Function, Immune Cell Trafficking	30	16

**Control v. Starved without scales (STWS)**			

	Cancer, Gastrointestinal Disease, Tumor Morphology	39	20
	Cancer, Reproductive System Disease, Genetic Disorder	30	16
	Lipid Metabolism, Molecular Transport, Small Molecule Biochemistry	26	14

**Starved (ST) v. Starved without scales (STWS)**			

	Cancer, Gastrointestinal Disease, DNA Replication, Recombination, and Repair	46	27
	Lipid Metabolism, Small Molecule Biochemistry, Vitamin and Mineral Metabolism	39	24
	Endocrine System Development and Function, Lipid Metabolism, Skeletal and Muscular System Development and Function	36	23

Score and number of molecules in each network are given.

### Most highly up-regulated genes: individual analyses

Analysis of the differentially expressed genes in each of the comparisons was restricted to up-regulated genes and those which could be assigned a putative function via the Uniprot/Swissprot and Uniprot/Trembl databases [37]. Nonetheless, one aspect to bear in mind is that approximately 50% of the oligoarray transcripts had no known match to any transcripts with functional annotation which limits the overall analysis and therefore the pathways invoked could only be inferred from those genes with a functional annotation. The unknowns will form an important aspect of future investigations, particularly those that are differentially expressed in more than one comparison (see Figure 2 and additional file 1). The following discussion focuses on the results of the samplings at 3 days.

### The effect of scale removal in fed animals

This initial analysis compared the most differentially expressed probes in the group of animals which had scales removed with control animals (Table 4). These probes shared high sequence similarity with genes involved in cell cycle regulation, cell proliferation and adhesion, immune response and antioxidant activities. Whilst many of the putative functions have been ascribed from human or mammalian research, both the receptor-transporting protein 2 and IFI56 have been identified in salmon and carp, respectively as interferon responsive genes induced in response to viral infections [38,39]. In addition Galectin 3 and LOC406638 have putative roles in the immune response, whilst methionine sulfoxide reductase and cytochrome p450 2W1 have antioxidant activities, indicating that removal of scales provoked an inflammatory response, with activation of cell defence mechanisms to protect the animal against the breach in external protection.

**Table 4:** "Top 20 known genes" up-regulated in the group without scales (WS) in relation to the control (N) on day 3.

Clone	LogFold	adj P-val	Acc. Number	Gene Identification	Putative function
SAPD03340	2.794	0.021	Q6P0E4	Type I cytokeratin, enveloping layer	Structural protein.

SAPD14269	2.453	0.001	Q9BUX1	Cation transport regulator-like protein 1	Potential component of the unfolded protein response.

SAPD07472	2.389	0.022	Q5QGT7	Receptor-transporting protein 2	Promotes functional cell surface expression of olfactory receptors, but also shown to be induced by interferon in response to infection.

SAPD04707	2.227	0.004	Q5QGT7	Receptor-transporting protein 2	Promotes functional cell surface expression of olfactory receptors, but also shown to be induced by interferon in response to infection.

SAPD01524	2.217	0.021	Q9BUX1	Cation transport regulator-like protein 1	Potential component of the unfolded protein response.

SAPD25017	2.008	0.000	NP_999926	Hypothetical protein LOC406638	GTP binding, immune function, specifically related to bacteria.

SAPD05261	1.934	0.016	Q7T2R0	IFI56	Immune function: antiviral effect.

SAPD23209	1.796	0.003	O43175	D-3-phosphoglycerate dehydrogenase	Amino acid biosynthesis, cell growth and differentiation, metabolic development and CNS function.

SAPD24645	1.771	0.006	P00480	OTCase Ornithine transcarbamylase	Amino acid biosynthesis.

SAPD22329	1.756	0.003	Q8TAV3	Cytochrome P450 2W1 EC 1.14.14.- CYPIIW1	Oxidative degradation and detoxification.

SAPD12115	1.752	0.024	Q08380	Galectin-3-binding protein precursor	Cell attachment and adhesion. May play a role in host defenses.

SAPD22251	1.735	0.006	Q6PGQ7	Protein aurora borealis (HsBora)	Cell division and mitosis.

SAPD15427	1.712	0.015	NP_932346	Methionine sulfoxide reductase B3 isoform 1	Antioxidant repair.

SAPD22138	1.582	0.004	Q16667	Cyclin-dependent kinase inhibitor 3	Haematopoietic ell cycle regulation.

SAPD25026	1.531	0.014	NP_001003640	Hypothetical protein LOC445246	RNA binding. Plectrin domain present in some forms of cytoskeletal muscle and ribosomal S10 protein (translation).

SAPD19262	1.418	0.008	Q13309	S-phase kinase-associated protein 2	Cell cycle progression/cell growth and apoptosis. Ubiquitination and degradation of proteins.

SAPD19571	1.394	0.015	O60566	Mitotic checkpoint serine/threonine-protein kinase BUB1 beta	Mitotic checkpoint protein.

SAPD17118	1.393	0.041	Q9UBR1	Beta-ureidopropionase (BUP-1)	Amino acid biosynthesis.

SAPD13849	1.390	0.021	Q9H7X7	Rab-like protein 5	Regulator of haematopoietic cells with roles in cell growth, survival, differentiation, cytokine production, chemotaxis, vesicle trafficking and phagocytosis.

SAPD21101	1.355	0.015	P06493	Cyclin-dependent kinase 1 (CDK1)	Key role in the cell cycle. Required for entry into S phase and mitosis.

The "Top 20 known genes" were taken from 36 transcripts of which 16 showed no match to genes of known function. Putative function was assigned via BLAST sequence similarity searching. All matches are in excess of 1.0 e^-10^. Definitions: LogFold = Estimate of the log2-fold change corresponding to the effect; adj p val = adjusted as described in methods.

During regeneration, the immune system is important for immune surveillance and control of pathogens, but there is increasing awareness of the importance of immune physiology. The latter term refers to the role of the immune system in tissue homeostasis and it is increasingly recognised that complement, lymphocytes and monocyte derived cells (MDC) promote tissue growth and regeneration in mammals (reviewed by [40]). This aspect has received little attention in fish, as research about immune functioning is generally focussed on infection or disease control, an important priority for aquaculture (see reviews by [41-43]). Immunological diseases and lymphoid tissue structure and development are an enriched category (IPA) for transcripts in fish with regenerating skin and scales. In addition to the probes listed in the tables for the different treatments, transcripts for chemokines (CCL3 and CCL4) associated with monocytes and macrophages were also identified and it remains to be established if their presence is associated with immune surveillance or immune physiology and tissue regeneration [44]. Recent investigations in stem cell biology have linked several molecules traditionally associated with the immune cells/function to stem cells. For example, immune associated transcripts differentially expressed in skin/scale from fasted fish 3 days after scale removal included CD55 (delay accelerating factor), a modulator of complement activity and a recently identified candidate surface marker for early and late definitive endoderm [45]. Similarly, CD200, a novel immunosuppressive molecule, has been identified as a biomarker of the hair follicle bulge in human and dog skin [46-48]. Future work will be required to establish the role of immune physiology and cellular defence mechanisms in the regenerating fish skin and also the involvement of stem cells in this process.

At the same time as the immune response, there is a clear requirement to rapidly re-construct this external barrier with various genes involved in metabolic processes such as amino acid biosynthesis and also cell division and proliferation. Interestingly, in a link with the IPA results, several of these genes have been described in cancer studies. Cyclin-dependant kinase inhibitor is involved in haematopoietic cell cycle regulation and has been shown to be over-expressed in breast and prostate cancer [49]; S-phase kinase-associated protein interacts with c-myc during the G1-S phase transition of the cell and is a co-factor of c-myc which is a known transcriptional regulator of oncoproteins and involved in cell growth, apoptosis and oncogenesis [50]; whilst the mitotic check point serine/threonine protein kinase has been shown to be preferentially expressed in cells with a high mitotic index [51]. Adaptation to new conditions involves an element of cytoskeletal re-modelling [52], as evidenced by the up-regulation of cytokeratin which has been associated with epidermis development, fibrinolysis and also regulation of angiogenesis. It is tempting to speculate that the up-regulation of cytokeratin in response to scale removal may represent a keratinization-like phenotype provoked by the osmotic shock. There was also up-regulation of genes involved in apoptosis such as Galectin-3 and the multifunctional S-phase kinase-associated protein (described earlier) and the somewhat confusingly named cation transport regulator-like protein [50,53]. Hence competing interests between infection/inflammation control and cellular proliferation/tissue repair in fish with scales removed appear to be ongoing.

### The effect of food deprivation with no scale removal

Skin tissue metabolism is clearly being redirected as the animals cope with adaptation to food deprivation. One aspect of this is a reduction in cell division with the up-regulation of angiopoietin-related protein 4 (ANGPTL4), MYND and KIAA0711, all of which have been shown to play roles in the inhibition of proliferation (Table 5) [54-56]. Another regulator of a proto-oncogene (MYC) is present in the form of ubiquitin carboxyl-terminal hydrolase and levels of MYC decline is response to intracellular stress signals [57]. Molecular signals of cell stress are also present with up-regulation of antioxidants (glutathione-S-transferase, methionine-R-sulphoxide reductase, alcohol dehydrogenase and cytochrome p450 2W1).

**Table 5:** "Top 20 known genes" up-regulated in the fasted group (ST) in relation to the control (N) on day 3.

Clone	LogFold	adj P-val	Acc. Number	Gene Identification	Putative function
SAPD06994	4.485	0.017	NP_001073454	Cardiomyopathy associated 5	Maintenance of structural integrity of muscle cells, involved in muscle biology and pathology.

SAPD01696	4.201	0.000	Q6B4J3	Alcohol dehydrogenase Class VI	Oxidoreductase, but also developmental regulation in medaka.

SAPD22431	3.956	0.006	IPI00015568	Homolog of Homo sapiens Protein KIAA0711	Transcriptional repression.

SAPD06947	3.919	0.038	NP_001019764	Zinc finger, MYND domain containing 17	Functions as a co-repressor. Control of proliferation.

SAPD25996	3.677	0.001	Q9BY76	Angiopoietin-related protein 4 precursor	Inhibition of proliferation, migration and tubule formation of endothelial cells. May exert a protective action on endothelial cells via endocrine action.

SAPD07090	3.525	0.013	Q96RU2	Ubiquitin carboxyl-terminal hydrolase 28	DNA damage response check point. Regulates myc which is involved in cell proliferation, growth and apoptosis.

SAPD09760	3.499	0.001	IPI00298828	Beta-2-glycoprotein I precursor	Blood coagulation and immune response.

SAPD06461	3.484	0.001	Q9BY76	Angiopoietin-related protein 4 precursor	Inhibition of proliferation, migration and tubule formation of endothelial cells. May exert a protective action on endothelial cells via endocrine action.

SAPD20351	3.415	0.000	IPI00298828	Beta-2-glycoprotein I precursor	Blood coagulation and immune response.

SAPD20440	3.230	0.000	NP_065109	Patatin-like phospholipase domain containing 2	Energy homeostasis. May play a role in response of organism to starvation, enhancing hydrolysis of triglycerides etc to be used in situations of energy depletion.

SAPD25836	3.176	0.001	Q9Y3D2	Methionine-R-sulfoxide reductase B2	Antioxidant repair.

SAPD14080	2.926	0.003	O75385	Serine/threonine-protein kinase ULK1	Involved in autophagy, induced by nutrient depletion to provide amino acids within cells.

SAPD02047	2.925	0.008	Q90278	Homolog of Carassius auratus Kainate receptor beta subunit.	Synaptic plasticity.

SAPD22329	2.511	0.000	Q8TAV3	Cytochrome P450 2W1 EC 1.14.14.- CYPIIW1	Oxidative degradation and detoxification.

SAPD10144	2.426	0.003	Q15119	2 Pyruvate dehydrogenase kinase isoform 2	Regulation of glucose metabolism.

SAPD23550	2.415	0.016	Q99972	Myocilin precursor Trabecular meshwork-induced glucocorticoid response protein	Obstruction of fluid outflow from trabecular network in the eye, but functions in other tissues unknown. Glucocorticoid response protein.

SAPD08799	2.392	0.002	IPI00328648	Homolog of Homo sapiens CD151 antigen	Involved in cellular processes including cell adhesion and migration.

SAPD08134	2.331	0.004	P28906	Hematopoietic progenitor cell antigen CD34 precursor	Cell-cell or cell-matrix adhesion. Role in early haematopoiesis.

SAPD06600	2.263	0.013	NP_060650	DEAD/H Asp-Glu-Ala-Asp/His box polypeptide 32	RNA metabolism, gene regulation.

SAPD01688	2.233	0.023	Q6PH41	Glutathione S-transferase, theta 3	Antioxidant, stress protein.

The "Top 20 known genes" were taken from 45 transcripts of which 25 showed no match to genes of known function. Putative function was assigned via BLAST sequence similarity searches. All matches are in excess of 1.0 e^-10^. Table definitions are the same as for Table 4.

During periods of food deprivation fish seem to maintain energy homeostasis, at least during the initial stages of fasting, by mobilizing energy reserves such as lipids and hepatic glycogen and reduction in the rate of glucose utilization and enhancement of lipid metabolism [31,58-60]. In fact, the genes differentially expressed in the array from the groups in which food was withheld suggests that lipid metabolism and angiogenesis are the main processes induced in the treated fish. Pyruvate dehydrogenase is involved in production of energy via glucose metabolism and ANGPTL4, in addition to a role in non-proliferation, and has also been shown to be a regulator of glucose homeostasis, lipid metabolism and angiogenesis [61,62], but this more conventional pathway may be supplemented by the actions of serine/threonine kinase ULK1 and a patatin-like gene. The former has been shown to be involved in autophagy induced by nutrient depletion to provide essential amino acids within cells, whilst the latter may enhance hydrolysis of triglycerides to provide free fatty acids to other tissues to be oxidised in situations of energy depletion [63,64]. Taken together the results appear to indicate that fish under food deprivation "slow down" their metabolism to save energy and break down macromolecules to release energy.

Interestingly, two of the genes putatively identified here play roles in human diseases, which may be of relevance to the condition of the fish in this experiment. Myospryn has been shown to be up-regulated in hypertrophy inducing conditions in humans and is involved in maintaining muscle integrity [65] and the phenotype of mutants of the CD151 antigen include fragility of the skin and mucus membranes [66]. Starvation directly affects muscle wastage in mammals and fish [67,68]. Hence these genes may be playing a similar structural role in fish as they do in humans, and represent novel candidates for understanding this physiological response in fish.

### The combined effect of food deprivation and scale removal

The most differentially regulated genes in this group of animals display a gene expression profile, which is intermediate between the previous two (Table 6 and Figure 2) with representatives of cell proliferation and cell cycle control genes, energy homeostasis, antioxidant repair enzymes and the immune response. The results of the gene expression profiles in this group clearly represent the whole organism trade-offs that are occurring within the fish for several competing essential cellular processes. Food deprivation leads to a reduction in metabolism, but if the animal is challenged, then there is the question of what predominates in terms of the minimal requirements for survival. Trade-offs occur [69] and a recent study in salmon clearly documents the competing transcriptomic responses to food deprivation and immune challenge [70]. Which requirements predominate in this study is difficult to determine and entail further studies. Perhaps, not surprisingly, there is an indication that repair processes are slowed under food deprivation with the enhanced presence of genes involved in blood coagulation and wound healing (Beta-2-glycoprotein I and lymphatic vessel endothelial hyaluronic acid receptor). To verify this hypothesis, further experimentation will be required with a more detailed sampling regime over the same or a slightly elongated time course with the same treatments.

**Table 6:** "Top 20 known genes" up-regulated in the group fasted without scales (STWS) in relation to the control (N) on day 3.

Clone	LogFold	adj P-val	Accession Number	Gene Identification	Putative function
SAPD06461	3.396	0.002	Q9BY76	Angiopoietin-related protein 4 precursor	Inhibition of proliferation, migration and tubule formation of endothelial cells. May exert a protective action on endothelial cells via endocrine action.

SAPD22431	3.138	0.031	IPI00015568	Homolog of Homo sapiens Protein KIAA0711	Transcriptional repression.

SAPD09760	2.742	0.007	IPI00298828	Homolog of Homo sapiens Beta-2-glycoprotein I precursor	Blood coagulation and immune response.

SAPD03340	2.530	0.039	Q6P0E4	Type I cytokeratin, enveloping layer	Structural protein.

SAPD20440	2.519	0.001	NP_065109	Patatin-like phospholipase domain containing 2	Energy homeostasis. May play a role in response of organism to starvation, enhancing hydrolysis of triglycerides etc to be used in situations of energy depletion.

SAPD22329	2.469	0.000	Q8TAV3	Cytochrome P450 2W1 (CYPIIW1)	Oxidative degradation and detoxification.

SAPD02465	2.422	0.016	Q6P2U4	Homolog of Brachydanio rerio Sulfotransferase family, cytosolic sulfotransferase 2	Metabolic action. Increases water solubility of compounds plus bioactivation of metabolites.

SAPD20351	2.364	0.006	IPI00298828	Homolog of Homo sapiens Beta-2-glycoprotein I precursor	Blood coagulation and immune response.

SAPD25836	2.176	0.017	Q9Y3D2	Methionine-R-sulfoxide reductase B2	Antioxidant repair.

SAPD24645	1.841	0.004	P00480	Ornithine carbamoyltransferase, mitochondrial precursor	Amino acid biosynthesis.

SAPD10144	1.793	0.033	Q15119	Pyruvate dehydrogenase kinase isoform 2	Regulation of glucose metabolism.

SAPD23209	1.724	0.004	O43175	D-3-phosphoglycerate dehydrogenase (3-PGDH)	Amino acid biosynthesis, cell growth and differentiation, metabolic development and CNS function.

SAPD22251	1.705	0.013	Q6PGQ7	Protein aurora borealis HsBora	Cell division and mitosis.

SAPD23370	1.704	0.012	Q9Y5Y7	Lymphatic vessel endothelial hyaluronic acid receptor 1 precursor	Facilitates cell migration during wound healing and inflamation.

SAPD14269	1.702	0.017	Q9BUX1	Cation transport regulator-like protein 1	Potential component of the unfolded protein response.

SAPD25026	1.689	0.007	NP_001003640	Hypothetical protein LOC445246	RNA binding. Plectrin domain present in some forms ofcytoskeletal muscle and ribosomal S10 protein (translation).

SAPD13849	1.589	0.005	Q9H7X7	Rab-like protein 5	Regulator of haematopoietic cells with roles in cell growth, survival, differentiation, cytokine production, chemotaxis, vesicle trafficking and phagocytosis.

SAPD16979	1.496	0.003	NP_998427	Eukaryotic translation initiation factor 5A	Translation.

SAPD19571	1.384	0.016	O60566	Mitotic checkpoint serine/threonine-protein kinase BUB1 beta	Control of the cell cycle.

SAPD21410	1.342	0.025	Q8WWL7	G2/mitotic-specific cyclin-B3	Control of the cell cycle.

The "Top 20 known genes" were taken from 37 transcripts of which 17 showed no match to genes of known function. Putative functionality was assigned via BLAST sequence similarity searching. All matches are in excess of 1.0 e^-10^. Table definitions are the same as for Table 4.

Curiously, one of the genes up-regulated in this group of animals, cytosolic sulfotransferase 2, which is involved in detoxification reactions, and participates in the activation and deactivation of hormones, neurotransmitters, steroids and bile acids [71], has been correlated with low plasma P levels in trout [72]. The preceding study developed several intestinal mRNA biomarkers for P depletion in the rainbow trout although only sulfotransferase 2 was modified in the present study presumably because the target tissue was different. The results of the present study in the sea bream suggest that cytosolic sulfotransferase 2 may be a promising general marker of P depletion in fish and certainly merits further investigation.

### The effect of scale removal under food deprivation compared with food deprivation

There was not such a pronounced effect as had been expected with scale removal and food deprivation and so an additional comparison was carried out between fasted animals and fasted animals with scales removed. Indeed, this comparison did produce the highest number of differentially expressed genes (429 up-regulated probes, compared with 53 (WS), 181 (ST) and 66 (STWS) when the latter three treatments were compared with control animals. Surprisingly little overlap in significantly up-regulated transcripts or modified networks were found between animals without scales and fasted animals without scales (see Tables, 3, 4, 7 and Figure 2A). The reason for this lack of overlap is difficult to explain but may result from asynchronous regeneration associated with the slow-down in cellular metabolism and activation of alternative pathways to ensure barrier function when food is in short supply. This comparison provides the clearest signal of the sea bream response to scale removal with half of the 20 most up-regulated annotated probes involved in cell division and mitosis (Table 7). These probes are ideal candidates for the monitoring of cell division processes related to the regeneration of scales. Of the remaining annotations, there are representatives of cell growth and metabolism (Interleukin enhancer binding factor, phosphoserine aminotransferase, D-3-phosphoglycerate dehydrogenase), cell proliferation (Macrophage stimulating 1 hepatocyte growth factor-like), and cell signaling (Inositol monophosphatase). The function of the multifunctional ubiquitin in the present experiments remains to be elucidated as this gene has a large number of roles [73] including cell cycle regulation, DNA repair, embryogenesis, regulation of transcription and apoptosis. Interestingly, this comparative analysis may reveal the first hint of the start of mineralization processes. In particular mutations in the gene 3-beta-hydroxysteroid-delta8, delta7-isomerase which catalyses the conversion of delta(8)-sterols to their corresponding delta (7) isomers is linked to chondrodysplasia punctata in humans (Conradi-Hunermann-Happle syndrome) that causes punctiform calcification of cartilage [74]. It remains to be established if this gene also influences the calcification process in fish but if it does it may represent a useful biomarker. Moreover, it suggests that up-regulation of transcripts involved in calcification occurs early in regeneration well before the most active phase of this process (estimated at 14-28 days in goldfish) [8].

**Table 7:** "Top 20 Known genes" up-regulated in the group fasted without scales (STWS) in relation to fasted group (ST) on day 3.

Clone	LogFold	adj P-val	Acc. Number	Gene Identification	Putative function
SAPD11916	4.104	0.009	IPI00005781	Splice Isoform A of Arachidonate 15-lipoxygenase, type II	Mediates allergic response. Restricts cell cycle progression.

SAPD06895	3.415	0.002	Q9Y617	Phosphoserine aminotransferase	Amino acid biosynthesis.

SAPD18045	3.355	0.000	Q02241	Kinesin-like protein KIF23	Mitosis and cell cycle.

SAPD00860	3.308	0.000	P14635	G2/mitotic-specific cyclin-B1	Control of the cell cycle at G2/M transition.

SAPD03340	3.298	0.006	Q6P0E4	Type I cytokeratin, enveloping layer	Structural protein.

SAPD11351	3.260	0.003	Q07426	Homolog of Carassius auratus Keratin	Structural protein.

SAPD04986	3.251	0.000	IPI00027157	Homolog of Homo sapiens CENP-F kinetochore protein	Involved in the cell cycle.

SAPD17903	3.160	0.000	IPI00412862	Homolog of Homo sapiens M-phase phosphoprotein 1	Involved in the cell cycle.

SAPD25367	3.039	0.001	Q5XFY1	Macrophage stimulating 1 Hepatocyte growth factor-like	Involved in cell proliferation and differentiation and tissue repair. Epidermal wound healing.

SAPD17933	3.017	0.000	Q15125	3-beta-hydroxysteroid-Delta8,Delta7-isomerase	Involved in sterol contribution to bone development.

SAPD23466	3.013	0.014	XP_641063.1	Hypothetical protein DDBDRAFT_0206057	Ubiquitin: multifunctional protein involved in cell cycle regulation, DNA repair, protein degradation, regulation of transcription, apoptosis and immune response.

SAPD20573	2.987	0.000	Q96EA4	Coiled-coil domain-containing protein 99	Involved in cell divsion via localisation of dyein and dynactin to kinetochore.

SAPD14492	2.975	0.001	P33981	Dual specificity protein kinase TTK	Associated with cell proliferation. Essential for the alignment of chromosomes by enhancing AURKB activity at centromere for mitotic check point.

SAPD09826	2.967	0.000	Q5T113	Uncharacterized protein C9orf156 Nef-associated protein 1	Hydrolyses acyl Co-A thioesters in vitro. Physiological function is unknown.

SAPD18060	2.898	0.001	NP_060445	Zwilch	Essential component of the mitotic check point. Required for dyein-dynactin and MAD1/MAD2 complexes onto kinetochores.

SAPD22251	2.847	0.000	Q6PGQ7	Protein aurora borealis HsBora	Cell division and mitosis.

SAPD11258	2.840	0.000	P29218	Inositol monophosphatase	Cell signalling.

SAPD18820	2.778	0.003	Q12905	Interleukin enhancer-binding factor 2	May regulate transcription of IL2 during T-cell activation: immune response. Functions as a ds RNA binding protein to promote autoimmunity.

SAPD23209	2.755	0.000	O43175	D-3-phosphoglycerate dehydrogenase 3-PGDH	Amino acid biosynthesis, cell growth and differentiation, metabolic development and CNS function.

SAPD18364	2.749	0.000	Q1LV50	Centromere protein P CENP-P	Component of the centromeric complex, involved in mitotic progression and chromosome segregation.

The "Top 20 known genes" were taken from 27 transcripts of which 7 showed no match to genes of known function. Putative functionality was assigned via BLAST sequence similarity searching. All matches are in excess of 1.0 e^-10^. Table definitions are the same as for Table 4.

The overexpression of developmental genes is already known to be involved in stem cell activation and in epidermal-dermal interactions. Examples such as FGFs, Wnts and SHH were not observed to be differentially regulated in the experiments described here, which could be for several reasons, including 1) some of those genes were not represented in the oligo array e.g. sonic hedgehog (SHH) and 2) the timescale of the experiment was not ideal to identify changes in gene expression of developmental genes associated with skin healing since by day 3 histological analysis revealed that epidermis is already re-established. Furthermore, metalloproteinases 2 (MMP-2) and 9 (MMP-9) have been recently suggested to have a role in scale regeneration in zebrafish [21]. However, MMP-2 is not represented in the sea bream microarray and although MMP-9 is represented (SAPD23115) it did not change significantly between the groups analyzed. Of the metalloproteinases present on the microarray (MMP-9, MMP-7.MMP-15 and MMP-28) only matrilysin (MMP-7) was modified and it was down-regulated in unfed fish without scales compared to the unfed fish on day 3 of the experiment. The sea bream oligo-array results were also queried for known calcitropic factors, for example, PTH (parathyroid hormone) and PTH related peptide (PTHrP), which are related with calcium and phosphorus homeostasis in fish [35,75], and calcitonin, whose hypo or hypercalcemic role in fish is not yet clarified [11,76,77], no significant differences in expression were observed. The same was observed for other calcitropic hormones represented in the array (such as prolactin, prolactin-receptor and PTH receptor 1), but to a certain extent this was to be expected given the previously estimated timescales for these processes, albeit in a different species [8]. Moreover, the target tissue in the present study, the skin, is not recognised as an important source of these hormones which tend to be produced in appreciable levels by specific endocrine tissues.

### Day 7 sampling

The biggest changes in the skin/scale transcriptome amongst the treated groups occurred at day 3. By day 7, when re-epithelisation had occurred and a thin regenerated scale was visible (Figure 1), relatively few differences were found when expression analyses were carried out. Over the four comparisons, a total of 49 probes were up-regulated only 21 of which had associated annotation, representing 17 putative unique transcripts (Table 8). It was also difficult to make generalisations about the on-going cellular processes, but the differentially expressed genes indicate a continued requirement for cell division and proliferation. The putative identification of the transforming acidic coiled coil 3 (TACC3) indicates the continuance of scale cell proliferation, as this gene in humans was shown to be involved in the control of cell growth and differentiation [78,79]. As in day 3, some genes are up-regulated in more than one of the comparisons made (Figure 2B). The GINS complex subunit 1 (PSF1 homolog) reported to be involved in regulating proliferation of stem cells, for example in response to acute bone marrow regeneration in mammals [80], is up-regulated in 3 of the four comparisons performed for both days 3 and 7 after scale removal where the factor analysed is the skin/scale regeneration. Taken together, the up-regulation of the GINS complex transcripts in all the groups where scales were removed at both day 3 and 7 of regeneration may indicate that, in teleosts, this transcript and its protein product may be regulating the process of skin/scale regeneration by the induction of cell proliferation.

**Table 8:** Known Genes up-regulated in the 7 day sampling.

Clone	LogFold	adj P-val	Acc. Number	Gene Identification	Putative function
**Group without scales (WS) in relation to the control (N)**					

SAPD11984	5.158	0.002	Q6E5T5	Claudin 1	Calcium-independent cell-adhesion activity.

SAPD02183	3.186	0.015	IPI00028413	Inter-alpha globulin Inhibitor H3	Hyaluronan metabolic processes.

SAPD18084	2.505	0.012	NP_001012506	Hypothetical protein LOC503524	Oxidoreductase. Potential role in ketone utilisation as a secondary energy source or generate precursors for lipid and sterol synthesis.

SAPD01661	2.356	0.019	Q5PSM1	Betaine homocysteine methyltransferase	Involved in methionine and betaine production (membrane composition, osmolyte concentrations and protein unfolding).

SAPD00429	1.129	0.013	Q14691	DNA replication complex GINS protein PSF1	Essential role in DNA replication.

SAPD11480	1.049	0.032	NP_001011729	Transforming acidic coiled coil 3	Involved in centrosome/mitotic spindle dynamics, gene regulation.

**Group starved (ST) in relation to the control (N)**					

SAPD06461	3.250	0.003	Q9BY76	Angiopoietin-related protein 4 precursor	Inhibition of proliferation, migration and tubule formation of endothelial cells. May exert a protective action on endothelial cells via endocrine action.

SAPD25996	2.942	0.011	Q9BY76	Angiopoietin-related protein 4 precursor	Inhibition of proliferation, migration and tubule formation of endothelial cells. May exert a protective action on endothelial cells via endocrine action.

SAPD10144	1.903	0.023	Q15119	Pyruvate dehydrogenase kinase isoform 2	Regulation of glucose metabolism.

SAPD22443	1.672	0.008	Q96DE5	Uncharacterized protein C10orf104	Cell cycle control.

SAPD13172	1.576	0.014	NP_001018326	villin 2 ezrin like	Involved the cytoskeleton, may influence rho signalling pathways.

**Group starved and without scales (7STWS) in relation to the control (7N)**					

SAPD06461	2.772	0.011	Q9BY76	Angiopoietin-related protein 4 precursor	Inhibition of proliferation, migration and tubule formation of endothelial cells. May exert a protective action on endothelial cells via endocrine action.

SAPD13675	2.203	0.029	P40313	Chymotrypsin-like protease CTRL-1 precursor	Protein degradation, degradation of old proteins for nutrition, control of protein activity, defense activity.

SAPD18879	1.437	0.007	P50570	Dynamin-2	Involved in the cytoskeleton and muscle structure.

SAPD00429	1.411	0.002	Q14691	DNA replication complex GINS protein PSF1	Essential role in DNA replication.

**Group starved and without scales (7STWS) in relation to starved group (7ST)**					

SAPD03340	3.815	0.002	Q6P0E4	Type I cytokeratin, enveloping layer	Structural protein.

SAPD01645	3.471	0.028	Q99895	Caldecrin precursor (Chymotrypsin C)	Protease activity and hypocalcaemic activity: decreases serum calcium.

SAPD03019	1.759	0.044	P35222	Catenin beta-1 Beta-catenin	Regulation of cell adhesion. Transcriptional co-activator via the Wnt pathway (development, cell proliferation and differentiation).

SAPD00618	1.469	0.007	NP_998168	Hypothetical protein LOC406276	Important role in the modification and proliferation of neurons.

SAPD00429	1.199	0.009	Q14691	DNA replication complex GINS protein PSF1	Essential role in DNA replication.

SAPD21979	1.121	0.037	P50454	Serpin H1 precursor	Essential in collagen synthesis.

The genes from the comparisons WS v. N, ST *v. *N, STWS v. N and STWS v. ST were taken from 8, 14, 17 and 10 transcripts of which 2, 9, 13 and 4, respectively, showed no match to genes of known function. Putative functionality was assigned via BLAST sequence similarity searching. All matches are in excess of 1.0e^-10^. Table definitions are the same as for Table 4.

There is also an element of cytoskeletal remodelling via the up-regulation of structural proteins (e.g. cytokeratin) and protein degradation. Similarly, as was noted with the fasted group (when compared with controls), there are a couple of genes up-regulated in sea bream, that in humans produce structural problems when defective. Mutations in Dynamin 2 produce abnormally large nuclei in skeletal muscle cells, resulting in muscle weakness [81], whilst serpin H1 is an essential chaperone in collagen synthesis, with deficiencies in humans resulting in the premature rupture of placental membranes [82]. As with the 3 days fasted vs. fasted without scales comparisons, at day 7 there are up-regulated transcripts potentially linked with mineralization. Such as, caldecrin precursor that exerts a hypocalcaemic activity, decreasing serum calcium, and may indicate that the fish is now actively mobilising calcium for scale mineralization.

### Real time RT-PCR

To corroborate the microarray data gene-specific qPCRs were performed. In general, the direction of change in expression was concordant between qPCR and the microarray probes and a positive Pearson correlation was obtained between qPCR and probe_1 (r^2 ^= 0.895, p < 0.0001 n = 40) and between qPCR and probe_2 (r^2 ^= 0.901, p < 0.0001 n = 40) (Table 9). Despite the good correlation observed between the gene expression analyzed by qPCR and the microarray data, a few exceptions were observed, the qPCR fold-change for SPP1 transcript in group 3ST; for ColVA2 transcript in group 3WS; for Col1A1 transcript for group 3ST and for p22phox transcript in group 3STWS vs. starved group was not correlated with the microarray fold-change. The latter is explained by the high variability found for this gene in different individuals in the experimental groups. The best concordance between qPCR and microarray data is achieved when the observed microarray fold change is between 2 and 7 [36], and in the present experiments the genes analyzed by qPCR which had fold changes closest to the lower limit had lower correlations with the microarray data.

**Table 9:** Comparison of Fold-change values from qPCR target genes and microarray in the 3 day sampling.

		WS			ST			STWS			ST vs STWS		
**SAPD ID**	**Target Transcript**	**qPCR**	**Probe 1**	**Probe 2**	**qPCR**	**Probe 1**	**Probe 2**	**qPCR**	**Probe 1**	**Probe 2**	**qPCR**	**Probe 1**	**Probe 2**

SAPD03530	Collagen V alpha2	1.087*	0.780	0.758	0.836	0.814	0.782	0.646	0.598	0.580	0.772	0.734	0.741

SAPD01318	Collagen I alpha1	0.838	0.398	0.547	1.294*	0.570	0.770	0.467	0.281	0.413	0.361	0.492	0.536

SAPD26722	SPP1	0.552	0.116	0.130	1.109*	0.415	0.477	0.501	0.293	0.323	0.451	0.708	0.678

SAPD00344	p22phox protein	0.574	0.255	0.250	0.367	0.296	0.303	0.380	0.231	0.231	1.035*	0.780	0.763

SAPD13946	Retinol-binding protein I	3.097	1.738	2.117	0.877	0.680	0.765	1.840	2.023	2.329	2.098	2.976	3.044

SAPD02730	Glutathione S-transferase A5	1.846	1.774	1.726	0.697	0.873	0.900	2.168	2.080	1.896	3.112	2.384	2.017

SAPD23304	Cytochrome c	1.373	1.874	1.949	0.625	0.873	0.845	2.149	2.180	2.278	3.438	2.498	2.696

SAPD20351	Beta-2-glycoprotein I	4.507	2.292	2.698	6.490	9.503	10.862	3.492	3.870	4.304	0.538	0.407	0.396

SAPD05261	IFI56	2.144	3.291	3.213	0.372	0.422	0.417	1.385	2.193	1.906	3.728	5.202	4.574

SAPD02155	pCNA	1.329	1.754	1.62	0.558	0.517	0.489	1.598	1.692	1.585	2.862	3.273	3.240

Comparisons were made for each treatment in relation to the control with the additional comparison of fasted vs. fasted without scales (ST vs. STWS). Values correspond to the Fold-change which was calculated by the ratio of mean expression values between treatment and control groups (individual biological replicates of n = 5 for both qPCR and microarray). * indicate where differences between qPCR and microarray data were observed.

## Conclusions

Fish skin is a very metabolically active organ which has crucial physiological functions, in osmotic regulation and is also an important immune barrier. Loss of scales and superficial wounds occur in both wild and captive teleosts and the vital importance of integument integrity means damage must be repaired as soon as possible. Although several studies exist which characterise tissue and cellular changes underlying skin regeneration in teleosts, molecular studies have largely been centred on scale formation and calcification, with the latter process not taking place until 14-28 days after scale removal [8]. The study described here, concentrates on the initial stages of scale removal and re-epithelialization. Our results show that this is a dramatic process, mainly occurring within the first three days after scale loss. The identification of numerous genes involved in the mitotic checkpoint and cell proliferation indicates that, with a more detailed time course experiment, this procedure represents a good model for understanding cell proliferation and control within the constraints of whole animal physiology. The utilisation of starvation as a treatment emphasised not only the essential processes underlying repair of the epithelia, but also the competing whole animal physiological requirements with regard to barrier repair, infection control and energy homeostasis. Comparison with human disease genes has identified several putative candidates in fish for the maintenance of cytoskeletal structure, which are of potential use in aquaculture and fish husbandry studies.

## Methods

### Fish

Juvenile sea bream (*Sparus auratus*) were maintained at the Centre of Marine Science (CCMAR) field station (Ramalhete, Faro, Portugal) in through-flow seawater tanks (2000L) at 17-21°C, 36% salinity and 12 h light and 12 h dark photoperiod for several weeks prior to the start of the experiments. The maintenance of fish and subsequent experiments complied with the Guidelines of the European Union Council (86/609/EU) and was covered by a group 1 licence (Direcção-Geral de Veterinária, Portugal). Behaviour and health of animals was monitored visually each day and no mortality occurred during the experiment.

### Experimental Design

**Figure 3:**
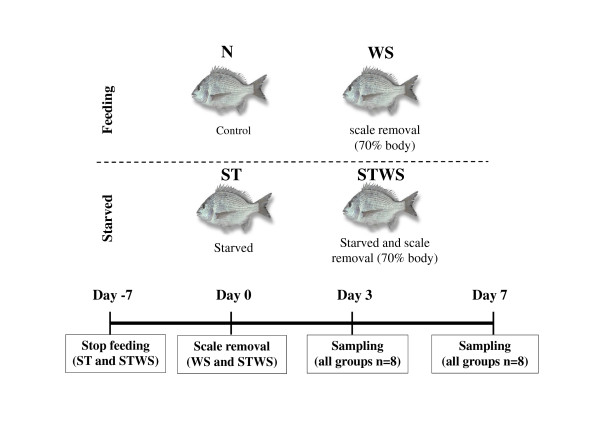
**Experimental design**. Eight groups were generated, four were sampled 3 days after scale removal and the others were sampled on day 7. Four different groups were created: N - control fish in normal conditions; WS - fish fed normal ration levels but with ~60-70% of scales removed; ST - fasted fish and STWS - fasted fish with ~60-70% of scales removed. Food was withdrawn from fish in the ST and STWS groups 7 days before the removal of the scales (day 0) and for the duration of the experiment.

Sea bream (n = 8 fish/tank, 93.4 ± 3.1 g) were acclimatised for one week to the experimental circuit which consisted of 8 through-flow seawater tanks (500L), with water maintained at 19-21°C, 36% salinity and a 12 h light and 12 h dark photoperiod. Food was withheld from the fasted experimental groups for 1 week (-7 days) prior to removal of the scales which was considered day 0 of the trial (Figure 3). The experiment had 3 treatment groups: ST = fasted for duration of experiment; WS = scales removed at time 0; STWS = fasted for duration of the experiment with scales removed at time 0 and the control group (N) with no treatment but subjected to the same anaesthesia/handling as the treatments groups.

Duplicate tanks for each treatment were prepared (day 3 and day 7) and 8 fish were sampled from one tank 3 days after the scales had been removed and from the second tank 7 days after the scales had been removed. Two tanks contained the control fish and were sampled at the same time as the experimental groups at day 3 and 7. To remove the scales, fish were lightly anaesthetised in 2-phenoxyethanol (1:5000, Sigma-Aldrich, Madrid, Spain) and scales were removed (approximately 60-70%) by wiping fish with a wet paper towel in order to minimize damage. For sampling fish were anaesthetised in 2-phenoxyethanol, as described above, weight and length was measured, blood collected and centrifuged (10,000 rpm for 5 minutes) and the plasma stored at -20°C. Fish were sacrificed by sectioning the spinal cord and skin was collected (approximately 1 cm^2^) from below the dorsal fin and carefully dissected free of muscle and snap frozen in liquid nitrogen and stored at -80°C. For histology, skin with some adhering muscle was fixed overnight at 4°C in 4% PFA.

### Plasma measurements

To determine the possible effect of treatments on plasma composition but also to evaluate the physiological condition of the experimental and control animals, calcium, phosphorus, glucose and lactate were determined. Duplicate samples of sea bream plasma (10 μl diluted 1/4 in milliQ water), collected from 8 fish/experimental group, 3 days after the removal of scales were measured. Colourimetric tests were performed according to the manufacturers' instructions and absorbance measured with a microplate reader:

• Calcium: Calcium-ο-C v/v kit (ο-cresolphtalein, v/v, colorimetric; Spinreact ref. 1001061, Spain), absorbance at 570 nm.

• Phosphorus: Phosphorus - UV kit (Phosphomolybdate, uv; Spinreact ref. 1001155, Spain), absorbance at 340 nm.

• Glucose: Glucose-TR kit (GOD-POD; Spinreact ref. 1001190/1001191/1001192, Spain), absorbance at 505 nm.

• Lactate: Lactate kit (LO-POD Enzymatic colorimetric; Spinreact ref. 1001330, Spain), absorbance at 505 nm.

### Histology of sea bream skin and scales

To characterize sea bream skin and scale morphology, samples fixed in 4% PFA were decalcified overnight in 0.5 M EDTA, pH 8, dehydrated through a graded ethanol series, saturated in xylene and impregnated and embedded in paraffin wax (Merck, Germany). Serial sections (5 μm) of skin were mounted on 3-aminopropyltriethoxysilane (APES; Sigma-Aldrich, Madrid, Spain) coated glass slides. The sections were dried overnight at 37°C, cooled to room temperature and stored or stained. To distinguish between collagen rich and/or mineralized and non-mineralized tissue, sections were stained with Masson's trichrome [83]. Skin sections were rapidly dehydrated through a graded series of alcohols, cleared in xylene and mounted in DPX mountant (BioChemika, Sigma-Aldrich, Madrid, Spain). Stained sections were analyzed using a microscope (Leica DM2000) coupled to a digital camera (Leica DFC480) and linked to a computer for digital image analysis.

### Sea bream microarray

A 4 × 44 k oligo-array developed and validated for the gilthead sea bream by Ferraresso *et al. *[36] was used in this study. The array contained 39,379 sea bream oligonucleotide probes covering 19,715 unique transcripts and of these, 19,650 were represented by two non-overlapping probes and 65 were present as a single probe. Owing to the expansion of teleost sequences in the public databases since the publication of Ferraresso *et al*. [36], a re-annotation of probes was carried out with Blastx similarity searches against the Uniprot/Swissprot and Uniprot/Trembl databases [37]. Annotation was assigned for probes with an expected score in excess of 1e^-10^.

Total RNA was extracted from five individual fish using an RNeasy Mini kit (Qiagen, Hilden, Germany) according to the manufacturer's instructions. RNA quality and integrity were checked using an Agilent 2100 Bioanalyser (Agilent technologies, Palo Alto, CA) and only samples with an RNA integrity index number (RIN) >7.5 were processed for use in microarray analysis. Samples from each treatment group (5 replicates) were labelled with Cy3-dCTP and hybridizations were performed using the Agilent One-Colour Microarray-Based Gene Expression Analysis protocol with the modifications described by Ferraresso *et al. *[36]. The arrays were scanned on an Agilent G2565BA DNA microarray scanner, at a resolution of 5 μm, and at two different sensitivity levels (XDR Hi 100% and XDR Lo 10%). The XDR Hi and XDR Lo images generated per array were analysed together and the data extracted. Background subtraction was performed using the standard procedure in the Agilent Feature Extraction Software 9.5.1. Spike-In Viral RNAs were used to control array hybridization intensities and ensure normalization gave a uniform signal across all microarray slides. The R [84] limma package [85] was used for microarray analysis. A factorial design of the treatments were compared by fitting a linear model [86] with differentially expressed clones selected by a Benjamini and Hochberg [87] globally adjusted p-value of 0.05 and a minimum two-fold change. The transcripts represented by two non-overlapping probes were only selected when both probes were differentially expressed.

The data discussed in this publication have been deposited in NCBI's Gene Expression Omnibus [88] and are accessible through GEO Series accession number GSE30717 (http://www.ncbi.nlm.nih.gov/geo/query/acc.cgi?acc=GSE30717).

### Gene Ontology Annotations and GO enrichment

Accession numbers associated with the probe annotations were used to assign GO and GOSLIM terms [89]. GO enrichment was determined by a proportion test between the number of clones representing a GO term on the array compared to the number of differentially expressed clones representing the same GO term in a given comparison with a p-value cut-off of 0.05.

### Ingenuity pathway Analysis

Cellular networks arising from the gene expression data were identified and established through the use of IPA (Ingenuity Systems, http://www.ingenuity.com). The sequences of differentially expressed genes from treatments collected at 3 days were all submitted to BLASTN in order to identify human orthologues. The accession numbers were extracted and used as identifiers in IPA together with the fold-changes of the corresponding differentially expressed genes. The Ingenuity knowledge base was used as a reference and direct and indirect relationships were included and no filters were applied. Bio functions, namely molecular and cellular functions and physiological system development and function significantly (p < 0.05) related with the input dataset were identified. Networks were then algorithmically generated based on their connectivity and a score was assigned. The score was used to rank networks according to how relevant they were to the genes in the input dataset.

### Microarray validation by real time RT-PCR

Array results were corroborated by real-time RT-PCR using when possible RNA extracted from the same individuals used for array analysis from all the different treatments at the 3 day time point. Ten genes were analysed and primers were designed using Beacon Design software (Premier Biosoft Int., Palo Alto, CA) (Table 10). For cDNA synthesis, 1 μg of total RNA was pre-treated with DNA-free Kit (Ambion, UK) to remove genomic DNA and then cDNA synthesis carried out using 250 ng of DNAse-treated total RNA, 200 ng of random hexamers (GE Healthcare, Little Chalfont, UK), 40 U of MMLV reverse transcriptase (RT) (Promega, Southampton, UK) and 5 U of RNAguard Rnase inhibitor (GE Healthcare, Little Chalfont, UK) in a final reaction volume of 20 μl. Q-PCR was performed in duplicate reactions using SYBRgreen chemistry (Power SYBR^® ^Green PCR Master Mix, Applied Biosystems, UK) and the relative standard curve method, using a StepOnePlus qPCR thermocycler and StepOne software v2.0 (Applied Biosystems, UK). PCR cycling conditions were 10 min at 95°C, followed by 55 cycles of 10 sec at 95°C, 20 sec at the optimal temperature for each primer pair (Table 10), and 30 seconds at 72°C. A final melting curve was carried out between 60 and 95°C for all genes and each produced single products/dissociation curves. Standard curves relating initial template quantity to amplification cycle were generated using serial dilutions of linearized plasmid DNA containing the gene of interest or of RT-PCR specific product obtained from the same specie and tissue, and the efficiency of qPCR reactions ranged between 82-100%, with the exception of SAPD20351 and SAPD13946 that had efficiencies of 73.1 and 78.6%, respectively, and all gave R^2 ^> 0.985. All amplicons were sequenced to confirm specificity of the PCR reaction. Ribosomal protein S18 (S18) expression was quantified using the same conditions as the other genes. No statistically significant differences were found between experimental groups so it was chosen as an endogenous reference gene to normalize qPCR data as it had a low inter-group variation and a similar level of expression to the analyzed genes. Statistical significance of relative gene expression between groups was analysed by one-way ANOVA using the software SigmaStat v.3.1 (SPSS Inc, Chicago, USA). Pearson correlations between the qPCR relative expression and microarray expression of both probes (average of individuals in each experimental group) were calculated for each gene. Statistical significance was established at p < 0.05.

**Table 10:** List of primers used for real time RT-PCR.

SAPD ID	Gene Name	Accession Number	Primer sequence (5'→ 3')	Amplicon (bp)	Temp (°C)	Efficiency
SAPD20351	Beta-2-glycoprotein I precursor	IPI00298828	F: TGGTTCGCCTCCTGTCTCC R: GGTTCTGGTGACTCATCCTCTG	178	60	73.1%

SAPD01318	Collagen alpha1 I chain precursor	P02452	F: AGACCTGCGTATCCCCAACTC R: GCCACCGTTCATAGCCTCTCC	110	57	83.4%

SAPD03530	Collagen alpha2 V chain precursor	IPI00293881	F: ACCTGTGACGACCTGAAGAGATGC R: TGGATGGGTTGGCGGAGATGC	145	60	86.8%

SAPD23304	Cytochrome c	P81459	F: AGGCATTCGTCCAGAAGTGTG R: TGGCATCGGTGTAGGAGTAGC	132	56	84%

SAPD05261	IFI56	Q7T2R0	F: ACCTCGCTGCTCAGTACCTC R: GCCTCCTCCGCCAAATCAATG	184	57	87.7%

SAPD02730	Glutathione S-transferase A5	Q7RTV2	F: AGACGTGCTGCTGCTTGAATGC R: TGGCTTCGGCTTCCTCTTGCTG	157	60	99.1%

SAPD00344	P22phox protein	NP_001027717	F: ATGCTTGCCACCGTCCTG R: TCTTGATGCTCTCTGCGACTG	139	60	82.4%

SAPD02155	Proliferating cell nuclear antigen (pCNA)	P12004	F: GAGCAGCTGGGTATTCCAGA R: CTGTGGCGGAGAACTTGACT	148	60	83.4%

SAPD13946	Retinol-binding protein I, cellular	P82980	F: TCCGCACCATAACCACCTTCAAG R: CCAGCCTCGTCCTTCCTTCTCC	168	60	78.6%

SAPD26722	SPP1 protein	NP_001002308	F: AGGTTGCTGACAGTTCTGAGAG R: GCGGCTGCTGCTACAATG	130	57	92.2%

**---**	Ribosomal protein S18	AM490061	F: AGGGTGTTGGCAGACGTTAC R: CTTCTGCCTGTTGAGGAACC	164	60	92.1%

Microarray Probe ID, primer sequences, amplicon sizes, annealing temperature and qPCR efficiency are shown.

## Authors' contributions

FAV designed and conducted the sea bream experiment, performed microarray analysis and qPCR for microarray validation, analysed the results and wrote the manuscript; SG assisted in the sea bream experiment and the oligo-array hybridization; SF and MM conducted the oligo-array hybridization; MAST performed the microarray data analysis; RC carried out the histology; LB conducted the oligo-array hybridization and edited the manuscript; MSC assisted in the interpretation of results and writing of the manuscript; AVMC designed the experiment and edited the manuscript; DMP designed and conducted the experiment, analysed the results and wrote the manuscript. All authors have read and approved the final manuscript.

## Supplementary Material

Additional file 1 **Complete lists of up or down-regulated genes in the microarray**. The differentially expressed probes of the microarray are listed for each of the comparisons made (N vs. WS; N vs. ST; N vs. STWS and ST vs. STWS) for both day 3 and 7 of the experiment. For each comparison two tables are presented: one for the up-regulated probes and another for the down-regulated probes. A summary of the information available is also given in the first spreadsheet of the file. Click here for file

Additional file 2 **GOSLIM (Biological process) diagrams**. The percentage of the GOSLIM categories is represented for all the transcripts in the microarray and also for the differentially expressed genes in the comparisons of day 3: N vs. WS; N vs. ST; N vs. STWS and ST vs. STWS. Click here for file

Additional file 3 **Top five Bio Functions identified for each comparison by IPA on day 3**. Bio Functions for each category (Molecular and Cellular Functions, and Physiological System Development and Function), the p-value and the number of implicated molecules are shown. Click here for file
