# Supplementary figures and images for: Skin healing and scale regeneration in fed and unfed sea bream, Sparus auratus
Source: BMC Genomics. 2011 Oct 7;12:490. doi: 10.1186/1471-2164-12-490 (PMC3199283; doi:10.1186/1471-2164-12-490)

### Array GOSLIM

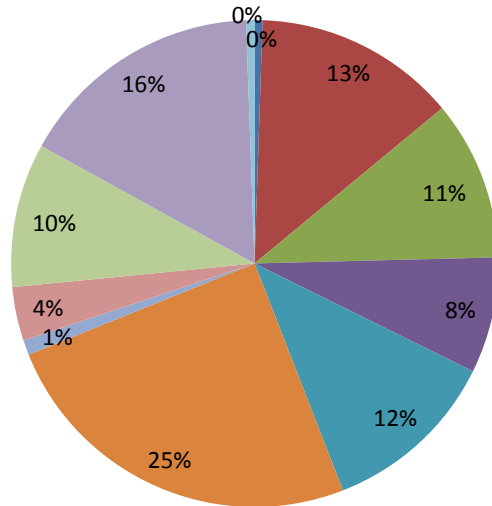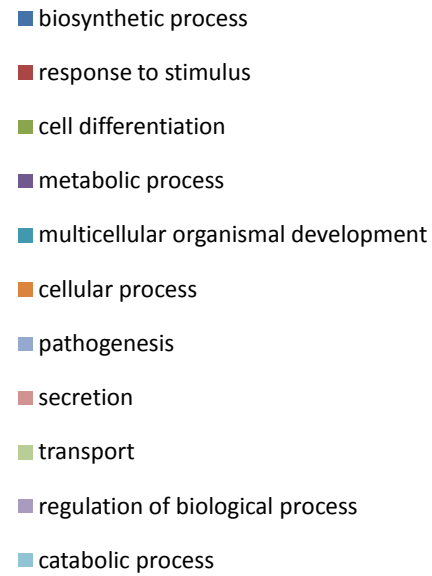

### N vs. ST (Day 3)

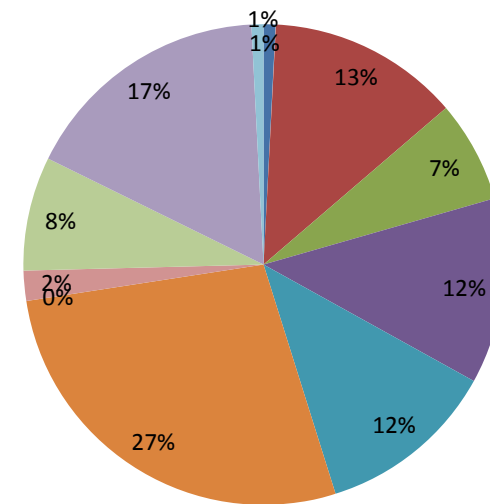

### N vs. WS (Day 3)

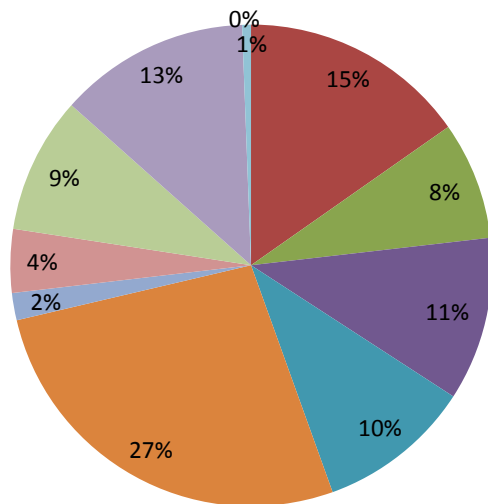

### N vs. STWS (Day 3)

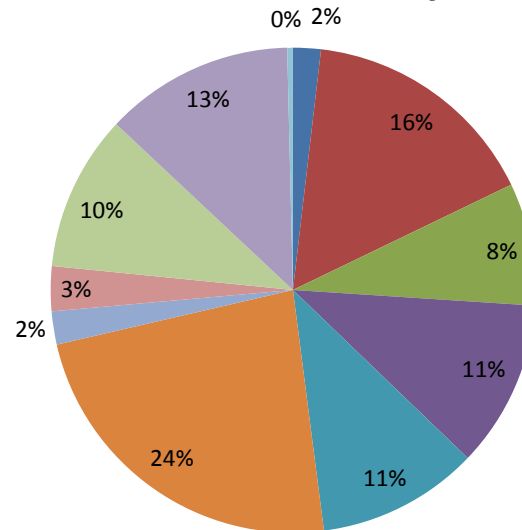

### ST vs. STWS (Day 3)

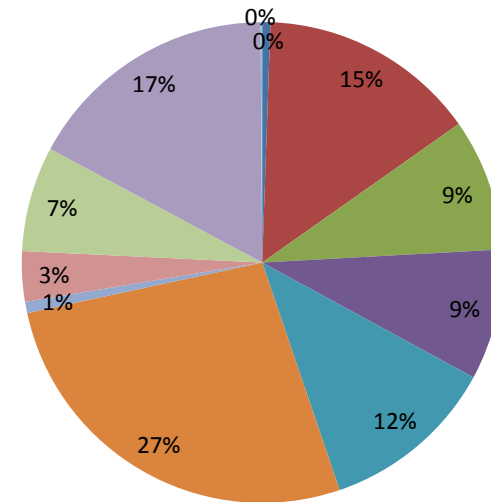

Supplement: Additional file 2 — GOSLIM (Biological process) diagrams. The percentage of the GOSLIM categories is represented for all the transcripts in the microarray and also for the differentially expressed genes in the comparisons of day 3: N vs. WS; N vs. ST; N vs. STWS and ST vs. STWS. [file 1471-2164-12-490-S2.PDF]
