# Supplementary material for: Skin healing and scale regeneration in fed and unfed sea bream, Sparus auratus
Source: BMC Genomics. 2011 Oct 7;12:490. doi: 10.1186/1471-2164-12-490 (PMC3199283; doi:10.1186/1471-2164-12-490)
Supplement: Additional file 3 — Top five Bio Functions identified for each comparison by IPA on day 3. Bio Functions for each category (Molecular and Cellular Functions, and Physiological System Development and Function), the p-value and the number of implicated molecules are shown. [file 1471-2164-12-490-S3.DOC]

**Additional File 3 – Top five Bio Functions identified for each comparison by IPA on day 3.** Bio Functions for each category (Molecular and Cellular Functions, and Physiological System Development and Function), the p-value and the number of implicated molecules are shown.

| **Group** | **Molecular and cellular** | **p-value** | **# Molecules** | **Physiological System Development and Function** | **p-value** | **# Molecules** |
| --- | --- | --- | --- | --- | --- | --- |
| **NvsWS3** |  |  |  |  |  |  |
|  | Amino Acid Metabolism | 7.17E-07 - 1.37E-02 | 8 | Lymphoid Tissue Structure and Development | 1.74E-04 - 6.89E-03 | 2 |
|  | Small Molecule Biochemistry | 7.17E-07 - 1.37E-02 | 15 | Tissue Morphology | 5.16E-04 - 1.37E-02 | 7 |
|  | Cell Cycle | 4.32E-05 - 1.37E-02 | 12 | Hematological System Development and Function | 7.53E-04 - 1.37E-02 | 8 |
|  | Cell-To-Cell Signaling and Interaction | 7.77E-05 - 1.37E-02 | 12 | Hematopoiesis | 7.53E-04 - 1.37E-02 | 6 |
|  | Post-Translational Modification | 1.74E-04 - 1.37E-02 | 5 | Connective Tissue Development and Function | 1.36E-03 - 1.37E-02 | 7 |
| **NvsST3** |  |  |  |  |  |  |
|  | Cell Cycle | 3.51E-05 - 4.75E-02 | 12 | Hair and Skin Development and Function | 2.10E-03 - 4.69E-02 | 3 |
|  | Lipid Metabolism | 1.55E-04 - 4.69E-02 | 12 | Tissue Development | 2.10E-03 - 3.67E-02 | 10 |
|  | Nucleic Acid Metabolism | 1.55E-04 - 4.18E-02 | 11 | Hematological System Development and Function | 5.21E-03 - 4.81E-02 | 6 |
|  | Small Molecule Biochemistry | 1.55E-04 - 4.69E-02 | 22 | Immune Cell Trafficking | 5.21E-03 - 3.01E-02 | 5 |
|  | Cellular Assembly and Organization | 1.67E-04 - 4.69E-02 | 8 | Cardiovascular System Development and Function | 5.32E-03 - 4.69E-02 | 6 |
| **NvsSTWS3** |  |  |  |  |  |  |
|  | Amino Acid Metabolism | 1.93E-06 - 1.50E-02 | 7 | Tumor Morphology | 1.49E-04 - 1.50E-02 | 8 |
|  | Small Molecule Biochemistry | 1.93E-06 - 1.50E-02 | 28 | Organismal Survival | 2.44E-04 - 7.13E-04 | 9 |
|  | Drug Metabolism | 7.90E-05 - 1.50E-02 | 6 | Lymphoid Tissue Structure and Development | 3.70E-04 - 1.00E-02 | 2 |
|  | Lipid Metabolism | 7.90E-05 - 1.50E-02 | 16 | Organ Development | 4.94E-04 - 1.50E-02 | 7 |
|  | Vitamin and Mineral Metabolism | 7.90E-05 - 1.50E-02 | 7 | Organ Morphology | 4.94E-04 - 1.50E-02 | 12 |
| **STvsSTWS3** |  |  |  |  |  |  |
|  | Cell Cycle | 2.81E-08 - 1.91E-02 | 55 | Hematological System Development and Function | 2.34E-04 - 1.87E-02 | 21 |
|  | Cellular Assembly and Organization | 1.88E-06 - 1.91E-02 | 43 | Immune Cell Trafficking | 2.34E-04 - 1.87E-02 | 21 |
|  | DNA Replication, Recombination, and Repair | 8.07E-06 - 1.91E-02 | 56 | Cardiovascular System Development and Function | 5.28E-04 - 1.91E-02 | 18 |
|  | Cellular Growth and Proliferation | 3.90E-05 - 1.91E-02 | 85 | Connective Tissue Development and Function | 7.20E-04 - 7.13E-03 | 12 |
|  | Cell-To-Cell Signaling and Interaction | 5.08E-05 - 1.91E-02 | 29 | Tissue Development | 8.20E-04 - 1.91E-02 | 26 |

Biofunctions significant at p<0.05.
